# Meiosis initiation: a story of two sexes in all creatures great and small

**DOI:** 10.1042/BCJ20210412

**Published:** 2021-10-28

**Authors:** Ieng Fong Sou, Rebecca M. Pryce, Wee-Wei Tee, Urszula Lucja McClurg

**Affiliations:** 1 Institute of Systems, Molecular and Integrative Biology, University of Liverpool, Liverpool L69 7ZB, U.K.; 2 Chromatin Dynamics and Disease Epigenetics Group, Institute of Molecular and Cell Biology, Agency for Science, Technology and Research (A*STAR), Singapore, Singapore; 3 Department of Physiology, Yong Loo Lin School of Medicine, National University of Singapore, Singapore, Singapore

**Keywords:** cancer, cancer testis antigen, meiosis, meiosis initiation, model organisms, TEX12

## Abstract

Meiosis facilitates diversity across individuals and serves as a major driver of evolution. However, understanding how meiosis begins is complicated by fundamental differences that exist between sexes and species. Fundamental meiotic research is further hampered by a current lack of human meiotic cells lines. Consequently, much of what we know relies on data from model organisms. However, contextualising findings from yeast, worms, flies and mice can be challenging, due to marked differences in both nomenclature and the relative timing of meiosis. In this review, we set out to combine current knowledge of signalling and transcriptional pathways that control meiosis initiation across the sexes in a variety of organisms. Furthermore, we highlight the emerging links between meiosis initiation and oncogenesis, which might explain the frequent re-expression of normally silent meiotic genes in a variety of human cancers.

## Meiosis in single cell organisms

Ultimately, the decision to enter into meiosis is vital for the creation of variance within species. therefore, meiotic entry is tightly regulated. Although fundamental characteristics of meiosis, such as the mechanics of homologous recombination, are highly conserved between species the decisive signals required to enter meiosis, and its regulation, are divergent in a variety of organisms.

**Figure 1.**
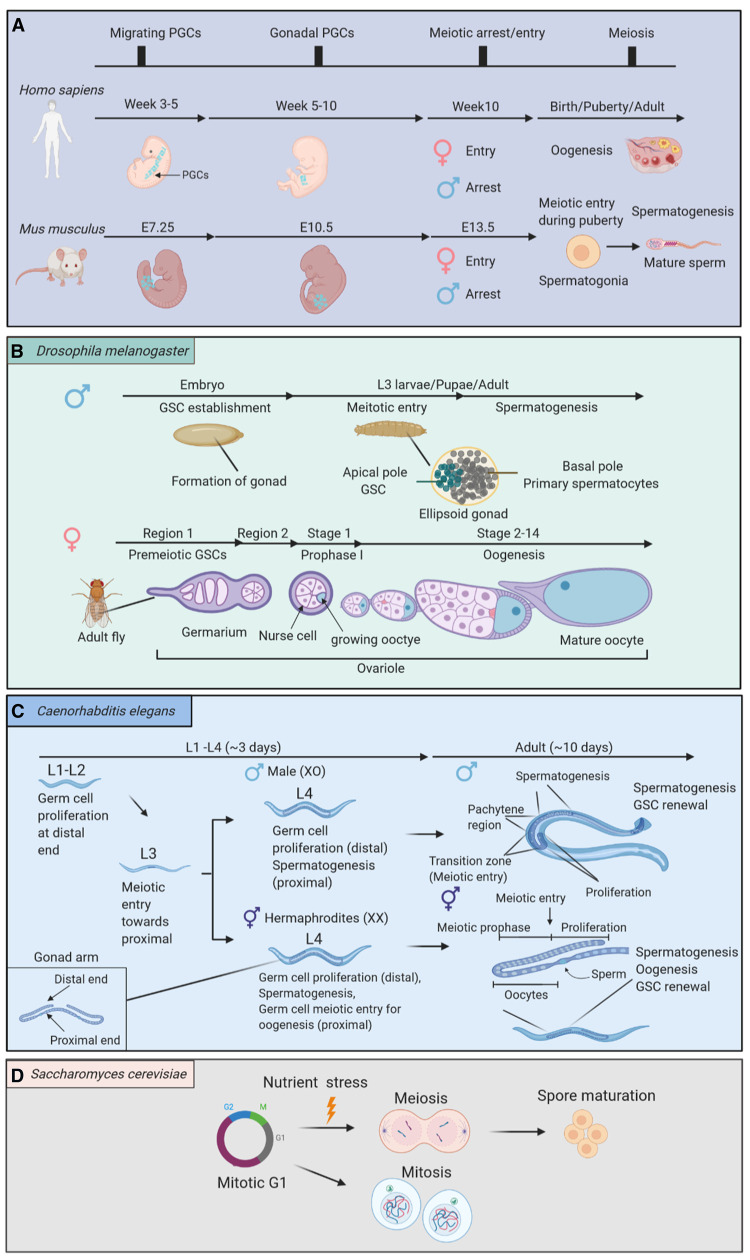
The timing of meiotic entry in distinct species and sexes. (**A**) In *Homo sapiens* and *Mus musculus*. Meiotic entry is largely conserved. In general, PGCs migrate through the midgut and arrive in the fetal gonads, where meiosis occurs in females and mitosis arrests in males. (**B**) In *Drosophila melanogaster* germ cells are derived from GSCs. Meiosis occurs in L3 larval instar stage in the male, GSCs shift from the apical pole to differentiate into primary spermatocytes in the basal pole, whereas GSCs enter prophase I within region 2 of germarium in adult female ovariole. (**C**) In *Caenorhabditis elegans* the dioecious hermaphroditic and male worms both enter meiosis during the L3 stage, GSCs shift from the distal to the proximal end as meiosis progresses. During L4 stage, male worms undergo spermatogenesis similarly to hermaphroditic worms but in hermaphrodites a subgroup of germ cells enter meiosis for oogenesis. With the renewal of GSCs, oogenesis and spermatogenesis can be maintained throughout adulthood. (**D**) *Saccharomyces cerevisiae* reproduces asexually upon nutrient starvation mitosis arrests at G1 and meiotic entry is triggered.

Single celled eukaryotes generally undergo meiosis only when ‘pressured’ to do so, with meiotic entry in both budding and fission yeast governed by relative nutritional availability. When growth conditions are optimal, mitosis is the default division process for single celled organisms since it enables rapid growth utilising the available nutrients. However, in response to nutrient-restriction, yeast signalling mechanisms lead to activation of meiotic programmes that have the ability to generate progeny that possess increased resilience to the newly applied pressure (Figure 1) [1]. Consequently, high concentrations of nitrogen and glucose can be considered inhibitors of budding yeast meiosis, which makes *Saccharomyces cerevisiae* one of the simplest organisms among laboratory models of meiosis. Budding yeast exist as both haploid and diploid with haploid yeast characterised by ‘a’ or ‘α’ mating type (MAT), mating produces a diploid (MATa/α) [2]. In response to nitrogen starvation or lack of fermentable carbon sources, diploid mitotic cells arrest at the G1 phase of mitosis and prepare for meiotic entry through global transcriptional activation of meiosis-associated genes [3,4]. Removal of nitrogen and glucose induces expression of Inducer of MEiosis 1 (IME1), a protein kinase master regulator of core meiotic proteins such as DMC1 and REC8 [1,5], which is repressed by Repressor of MEiosis 1 (RME1) in haploid cells. Consequently, haploid cells are not capable of responding to environmental cues and only MATa/α diploid cells can undergo meiosis as the complex formed by a1–α2 is able to repress expression of RME1 [6–8]. Nutritional regulation of IME1 expression in diploid cells is executed by Protein Kinase A (PKA) and Target Of Rapamycin Complex 1 (TORC1). In the presence of glucose, when cAMP levels are high, the PKA pathway is activated by the G-protein α subunit GPA2 allowing it to phosphorylate SOK2, which in turn acts to repress IME1 expression. Furthermore, activation of TORC1 through nitrogen availability sensing, also represses IME1 [9]. Upon nutrient withdrawal, reduced activity of the PKA pathway allows for IME1 expression and increased activity of the RIM11 kinase. RIM11 phosphorylates UME6 and IME1, which are then able to form a stable complex leading to the expression of early meiotic genes including the serine/threonine kinase Inducer of MEiosis 2 (IME2) [10]. This provides a negative feedback loop allowing cells to control meiotic timing as IME2 can lead to IME1 proteasomal degradation [11]. This flexible phosphorylation-dependent meiosis-mitosis switching mechanism provides yeast with survival and growth advantages, by ensuring usage of available nutrients and generation of new gametes which might gain the ability to survive ‘harsh’ environments [12].

Interestingly, as a consequence of adapting to different growth environments fission yeast appear to lack an IME1 orthologue. Meiotic initiation is instead governed by STE11^+^ with expression controlled by down-regulation of TORC1 and PKA signalling, similarly to IME1 in budding yeast. However, the main driver of meiosis initiation is not lack of glucose but rather nitrogen starvation [13]. Nitrogen limitation activates expression of STM1^+^, a GPA2^+^ inhibitor. Once expressed STM1^+^ leads to STE11^+^ expression regardless of glucose levels [14]. Further up-regulation of STE11 after conjugation occurs as diploids deactivate the PAT1 kinase through a decrease in phosphorylation [15]. This facilitates the activation of early meiotic genes such as MEI2^+^, PAT1 down-regulation is critical for MEI2^+^ activity. When growth conditions are optimal PAT1 phosphorylates MIE2 and STE11 leading to proteasomal degradation of MEI2 and STE11Rad24 binding which inhibits STE11 transcription factor activity.

## The timing of meiotic onset in multicellular organisms

The nutrient restriction requirement for meiosis initiation in *S. cerevisiae* and *S. pombe* is well characterised and readily replicated within the laboratory, making yeast a useful tool in meiotic research. However, multicellular organisms have an added layer of complexity as meiosis cannot be activated synchronistically in every cell of the organism and as such a complex system of regionalising meiosis, and consequently meiosis initiation, has developed. Consequently, in multicellular organisms specialised germline stem cells (GSC), which arise during embryogenesis, can divide to renew the GSC pool and differentiate into sex-specific germ cells that undergo meiosis [16]. However, the timing of meiotic initiation, as well as transcriptional and translational changes during meiosis, differ significantly between the sexes. In this section, we will discuss how organisation of meiosis initiation differs between the common multicellular models of meiosis by comparing and contrasting the context of meiosis initiation, its timing and germ cell origins.

Due to its regionalised nature, meiosis initiation in multicellular organisms depends on the migration of germ cells, highlighting the need for evolutionarily conserved mechanisms to facilitate germ cell development. In *Drosophila melanogaster* males and females exist as separate organisms and future gametes arise from GSCs in the testis and ovaries, respectively. These GSCs are differentiated from a subgroup of Primordial Germ Cells (PGCs) during the embryonic stage [17,18]. In contrast with humans, meiosis initiation in the fruit fly occurs much earlier in males than in females (Figure 1). In male flies, entry into meiosis and spermatogenesis is established in L3 larval stage within the ellipsoid gonad, where GSCs reside in the apical pole and differentiate into the primary spermatocytes within the basal pole [19]. At the apical tip of the testis resident hub cells divide mitotically and are crucial for GSC maintenance. Each GSC is surrounded by two cyst stem cells. GSCs divide asymmetrically to sustain the GSC pool and produce a gonialblast cell (GB). The GB cell remains associated with the cyst cells. GB divides mitotically, driven by Bam signalling, as in females [18,20]. Bam levels peak at the 8-cell M stage and these 8 cells enter their final division before meiotic onset [21]. The 16-cell cyst containing spermatogonia undergoes a final division to produce 64 spermatids. Mature gamete production continues throughout adulthood as males maintain a nice of 6–12 germ stem cells allowing testis homeostasis [19,22].

In female *Drosophila*, the GSC niche is established in L3 larval stage within the female gonad [23]. Meiosis occurs within the adult germarium located at the tip of the adult ovariole, which consists of three regions [24]. When a GSC divides, one of the daughter cells is driven further away from the cap cells due to spatial restriction. This cell is known as a cytoblast. In *Drosophila* proximity to bone morphogenic protein (BMP) signalling prevents cell differentiation through repression of Bam (bag of marbles), similarly to distal tip cell signalling that prevents meiotic entry in *C. elegans*. Once Bam is no longer repressed by BMP expression in the cytoblast (CB), it becomes a major driver of GSC differentiation [25]. Differentiated cytoblasts are able to divide mitotically until Bam levels peak before the final mitotic division producing a 16-cell cyst, of which just a single cell will progress through meiosis to become an oocyte. Whereas Bam is necessary in female flies for initial GSC differentiation as well as CB divisions, it is needed only for subsequent gonialblast division in males. In contrast with mammals, *Drosophila* females maintain a 2–3 GSC niche throughout adulthood giving them the ability to produce oocytes throughout their lifetime. Fly germ cell development is similar to mammals, since it is supported by surrounding somatic cells to provide essential cellular signalling molecules for germ cell maturation, leading to downstream transcriptional activation and subsequent embryonic development.

In contrast with *Drosophila*, the multicellular model worm *Caenorhabditis elegans* has the ability to produce both types of gamete within a single organism. Furthermore, the gonads are easily visible due to a transparent body, and therefore cytological analysis is possible on the whole organism, as well as on a single cell level. Experimentally manipulatable meiotic cells are in great supply with 50% of the total nuclei belonging to the germline [26], making *C. elegans* an incredibly useful tool for meiotic research.

Germline sex determination in *C. elegans* is controlled by a network of >20 genes. *C. elegans* possess both male and female reproductive organs. However, they can still produce male worms (designated X0) with a single X chromosome originating from the male germ cells, although this occurs at a very low frequency (<0.2% of total). The male phenotype results from the mis-segregation of X chromosomes in oocytes which can lead to oocytes with no X chromosome, which are then fertilised by sperm [27]. However, the vast majority (99.8%) of *C. elegans* are hermaphrodite (XX) worms which can self-fertilise and are able to perform both oogenesis and spermatogenesis [27]. In *C. elegans*, similarly to *Drosophila*, mitosis-to-meiosis transition is determined in a spatiotemporal manner. In *C. elegans* meiocytes progress through meiosis as they move away from the distal tip of the gonad. Germline stem cells are located at the distal gonad tip, surrounded by the distal tip cell (DTC). The DTC is adjacent to a region known as the progenitor zone, which contains a mixed population of mitotically dividing cells organised into population pools, beginning with GSC, followed by mitotically dividing cells and ending with meiotic S phase cells [28]. At least three complementary signalling pathways promote meiotic entry in worms: the RNA-binding protein GLD-1 (Germline Defective-1), GLD-2, and the recently discovered SCFPROM1 ubiquitin ligase complex. GLD-1/Notch signalling acts to inhibit the translation of mitotic cell cycle proteins, whereas the GLD-2 pathway leads to the translational activation of meiotic entry proteins. The SCFPROM-1 complex promotes degradation of mitotic cell cycle proteins [29]. The DTC releases GLP-1 activating ligands which promote proliferation of GSC and inhibit both GLD-1 and GLD-2 pathways repressing meiotic entry [30]. In *C. elegans*, germ cell amplification within the distal end is caused by interactions with sheath cells (sh1) during the L1–L2 stage until elongation of the gonad; consequently germ cells migrate further away from the distal end and are no longer controlled by GLD-1 signalling, which leads to meiotic entry [31]. Following the loss of GLP-1 (abnormal Germ Line Proliferation 1) signalling, mitotic cells complete their final division and enter meiosis, suggesting that GLP-1 is acting as a repressor of meiosis [32]. Thus, meiotic entry in *C. elegans* is controlled by spatiotemporal movement away from GSCs and the change in external signalling is associated with this movement. To ensure that hermaphrodites can self-fertilise, entry into male meiotic prophase I occurs in late L3 stage and mature sperm can be observed during L4 stage. However, germ cells that enter meiosis from mid-L4 onwards are differentiated into oocytes throughout adulthood [33]. In rare male worms, germ cells enter pachytene during mid L3 and undergo spermatogenesis from mid L4 and throughout adulthood (Figure 1) [33].

## Regulation of female meiosis in *Xenopus laevis*

One of the major difficulties associated with meiotic research lies in appreciating the differential regulation between male and female meiosis. Vertebrae female meiosis presents the additional challenges of prolonged duration, ethical issues and limited access to experimental material. The *Xenopus laevis* model system has been instrumental in studying the later stages of female meiosis due, in part, to the large size of the mature egg (∼1 mm diameter) and an ability to manipulate its contents via both immunodepletion and microinjection of experimental biomolecules and the generation of cell-free nuclear extracts, *ex vivo* [34]. The latter is possible because eggs contain a reservoir of RNA and proteins allowing for 12 rounds of transcription-free cell division following fertilisation prior to the onset of zygotic transcription after the Mid-Blastula transition (MBT). These early divisions are driven by cyclical translation and degradation of cyclin B, consequently the *Xenopus* system was critical for the purification of the biochemical drivers of meiotic and mitotic cell cycles [35]. During oocyte development, cells are synchronously arrested in the first meiotic metaphase until meiotic maturation is triggered by progesterone, which can be recapitulated *ex vivo* after manual oocyte dissection from the ovary or after egg laying is induced with hCG (the origin of the use of *Xenopus laevis* for the detection of human pregnancy; the ‘Hogben’ Test). Meiotic cell cycle arrest is facilitated by high endogenous cAMP levels and a decrease in cAMP induces inhibition of the cAMP dependent protein kinase (PKAc), which is necessary for ending meiotic arrest [36] through modulation of the PKA substrate ARRP19 [37], a key regulator of PP2A, which also has critical roles in later mitotic cell cycles [38]. Following completion of meiosis I, germinal vesicle breakdown (GVBD) proceeds the extrusion of the first polar body after which oocyte enters meiosis II without an intervening interphase and arrests at metaphase of the second meiotic division until fertilisation. MPF (maturation promoting factor), which is composed of cdc2 and B-type cyclins, is also critical for the G2-M transition [35,39] MPF activity is initiated by Mos, which activates the classical MAPK pathway, after which it is regulated in a cell cycle dependent manner by the CSF (cytostatic factor) [40]. Due to historical advantages of *Xenopus laevis* for developmental biology research [41], work has focused on the later stages of oocyte meiotic progression rather than early meiosis initiation, and will not be discussed further here. The *Xenopus* system and cell cycle are extensively reviewed in [34,42,43].

## Spermatogenesis and oogenesis in mammals

Male and female gametes are distinct in size, shape and their relative contribution to the fertilised embryo. Cytoplasmic and nuclear contents in the sperm are reduced as the oocytes contribute to all the essential organelles for the future zygote and are metabolically active, whereas sperm cells contribute to the future zygote by delivering the paternal genome, two centrioles, oocyte activation components and transcripts essential for embryonic development [44,45]. Therefore, optimal (reduced) size and innate motility have been prioritised in order to reach the egg. Consequently, the oocyte requires a longer maturation period compared with the process of spermatogenesis. During oogenesis, the centrosomes are eliminated [46] and centrioles within the future zygote are provided by mature spermatids [47]. Therefore, meiosis is regulated differently in the context of meiotic entry, timing, hormonal changes and outcomes during oogenesis and spermatogenesis in order to produce high quality gametes that have contrasting roles prior to fertilisation.

In mammals, meiosis takes place after the migration of primordial germ cells (PGCs) to the developing gonads, followed by differentiating into oogonia and spermatogonia, which are the precursors of egg and sperm, respectively. PGCs differentiate into mature gametes in a specific organ, spermatogenesis in male testis and oogenesis in female ovaries. In humans, PGCs travel to the developing gonads through the midgut during week 3 to 5 of embryogenesis. Female oocytes enter meiosis at week 10 during fetal development, while male germ cells undergo mitotic G1 arrest until puberty, when meiosis is initiated (Figure 1) [48]. Regulation of meiotic entry and progression in mice is very similar to that in humans. In mice, PGCs migrate to the fetal gonad at embryonic stage E10.5 and enter meiotic prophase I in female when male germ cells commit to mitotic arrest at E13.5 (Figure 1) [49]. Similarly, to lower class species such as worms and flies, mammalian germ cells also require sex-specific somatic cells to support different stages of germ cell development.

Male meiosis is initiated by major male sex hormones such as pituitary-secreted follicle stimulating hormone (FSH), luteinizing hormone (LH) and testosterone, which surge upon puberty [50]. In the testis, somatic supporting cells such as Sertoli and Leydig cells regulate spermatogenesis in an endocrine manner [50]. FSH acts on the Sertoli cell receptors while LH stimulates Leydig cells to secrete testosterone [51]. In concert with testosterone, FSH plays roles in providing essential factors and nutrients for male germ cell maturation [50]. These signals are required for spermatogonia to differentiate into primary spermatocytes allowing division into haploid secondary spermatocytes during meiosis I. Finally, spermatocytes differentiate into mature haploid spermatids within the seminiferous tubules of the adult testis [52]. To produce mature gametes throughout life, a proportion of spermatogonial stem cells (SSCs) can be renewed and differentiate, providing a continuous supply of sperm cells [53]. In spermatocytes, transcription is robustly activated from pre-meiotic (spermatogonia) until post-meiotic (round spermatid) stages [54]. At the final stage of male meiosis, a wave of transcriptional activation is required for round spermatids to prepare for the dramatic morphological change into mature sperm, including replacement of histones with protamines, flagellar formation and cytoplasmic removal [55]. These features are required to reshape the nucleus, resulting in dramatic reduction in the nuclear volume and a complete cessation of transcriptional activities during nuclear compaction [55,56]. Consequently, the final stages of spermatogenesis are characterised by condensed chromatin and depend on stored paternal mRNAs to generate fertilization-competent spermatocytes [55].

## Female PGCs differentiate into oogonia after arriving in the genital ridge and enter primary oocytes

In contrast with males, the number of female germ cells is limited with meiosis paused at different stages. Oocyte dictyate arrest in prophase I and oocyte maturation is supported by a group of somatic cells within the follicle known as granulosa cells, which wrap around the oocyte and provide a favourable environment to regulate maturation processes including growth, meiotic pausing and resumption. Female meiosis initiates in fetal ovaries followed by arrest at diplotene stage of prophase I caused by granulosa-secreted cyclic GMP (cGMP), propagated into the oocytes through gap junctions. During puberty, LH from the pituitary gland binds to the receptors on the granulosa cells resulting in a dramatic reduction in cGMP within the oocyte, which in turn resumes meiosis, resembling the GPCR-coupled nutrient starvation response present in yeast [57]. LH stimulation also leads to the breakdown of an oocyte-specific nuclear membrane called the germinal vesicle (GV), which marks the resumption of prophase I [58]. Oocyte maturation is facilitated by a group of cyclin-dependent kinases and cyclins including CDK1, CCNB1 and CCNB2 within the maturation promoting factor (MPF) complex [59]. At the end of meiosis I, homologous chromosomes are segregated into the secondary oocyte that proceeds into meiosis II and the first polar body, a small meiotically incompetent oocyte. At metaphase II, another arrest occurs in the secondary oocyte. This arrest is maintained by the cytostatic factor, which stabilises MPF activity and prevents cyclin B degradation induced by the elevation of Ca^2+^ levels when fertilisation occurs by fusing with the sperm [60]. Fertilisation terminates meiosis II, resulting in the protrusion of the second polar body and the mature fertilised ovum (now known as the zygote), which gives rise to the next generation [58]. Developing oocytes begin to acquire meiotic competence and become transcriptionally inactive until zygotic gene activation (ZGA) [61,62]. Oocyte maturation relies on stored dormant maternal transcripts that are generated during the primordial phase and translational control of these mRNAs is hence pivotal for oocyte maturation and early embryonic development [63].

## Regulation of mammalian meiotic initiation

Meiotic entry is governed by numerous, intricate signalling mechanisms, a few of which are discussed above. The essential signals that propagate from the embryonic gonad to the PGCs for promoting gametogenesis have been exclusively reviewed by [48] and [64]. In mammals, typically, FSH stimulates meiotic entry and induces the production of all trans retinoic acid (RA) to facilitate germ cell differentiation. RA is a vitamin A derivative that binds to nuclear RA receptors (RAR) and retinoid X receptors (RXR), activating transcription of RAR and RXR target genes. Consequently, RA has historically been considered to be the master regulator of meiotic entry in both oogenesis and spermatogenesis, although the precise signalling mechanism may be species-dependent [65]. A body of published studies demonstrates that RA driven activation of STimulated by Retinoic Acid gene 8 (*Stra8*) is crucial for meiotic onset [66–69]. Although STRA8 is normally cytoplasmic, it localises to the nucleus during meiotic onset where it works as a transcription factor utilising its basic helix loop helix (bHLH) domain. In the nucleus STRA8 binds promoters of multiple meiotic genes while also acting on its own promoter in a feedback loop [70]. However, STRA8 is not the only transcription factor required for meiosis initiation. SOHLH1, another bHLH containing transcription factor, interacts with STRA8 to activate some of the early meiotic genes crucial for both male and female meiosis initiation as well as synaptonemal complex formation and homologous recombination [71–73]. Furthermore, more recently, MEIOSIN was reported to be a direct downstream target of RA signalling [74].

RA is synthesised by three major retinaldehyde dehydrogenases (RALDH 1, 2, 3), in which RALDH2 and 3 are known to be the major sources of RA [65]. However, the mechanisms of STRA8 driven meiotic initiation remain rather ambiguous. Teletin et al. [65] reported that *STRA8* was expressed in the spermatocytes independently of RA signalling despite *RALDH2* and *RALDH3* being depleted. Similar results were observed in murine fetal ovaries [75], indicating that RA may not be indispensable for *STRA8* expression, or perhaps that RALDH1 is the favourable source of RA for meiotic entry [76]. In the fetal ovary meiotic entry is regulated primarily by RA signalling. However, Bone Morphogenetic Protein (BMP) signalling is also crucial for meiosis-specific transcriptional regulation [77], which is established in the PGCs. Within this network, ZGLP1, a direct downstream effector of BMP4 signalling, is able to switch on repressed bivalent genes that contribute to entering oogenesis [78]. Moreover, STRA8 can be activated by BMP2 expression as a downstream effector of Wnt/β-catenin signalling, which regulates timely initiation of meiosis in female PGCs [79,80].

Conversely, mitotic division in male PGCs halts at E13.5 and meiosis is delayed until puberty. Meiotic inhibitors such as NANOS2 and DMRT1 maintain male germ cells in mitotic arrest stage and prevent them from entry into meiosis [81,82]. Recently evidence has emerged that NANOS2 is a major regulator of initiating and maintaining mitotic arrest in a post-transcription regulation manner [83]. Furthermore, delays in male meiosis can be imposed by multiple RA repressors in order to prevent pre-mature meiotic onset. For instance, *FGF9* signalling plays a role in inhibiting *STRA8* expression in mice [84]. Moreover, a metabolising enzyme CYP26B1 expressed in Sertoli cells within the fetal testis degrades RA [85]. Consequently, upon puberty, DMRT1 was found to be diminished during zygotene and pachytene in human adult testis [82]. Similarly, *CYP26b1* is down-regulated in Sertoli cells prior to the onset of meiosis [86], followed by the expression of meiotic markers including DMC1, STRA8 and SYCP3 and the reduction in pluripotent markers within the adult testis, which mark meiotic entry.

Interestingly, although meiosis initiation is regulated by multiple signalling pathways within multicellular organisms, it has been reported that nutrient restriction and RA stimulation can activate the meiotic program through activation of a set of key transcription factors *in vitro* [87]. This observation is reminiscent of meiosis initiation in yeast, which is primarily nutrient and metabolism-driven. Expression of IME1 upon nutrient starvation in yeast triggers the activation of meiotic genes such as Spo11, Hop1 (HORMAD1 in human) and Dmc1 [88], which are also major meiotic regulators of double strand break formation, a crucial step for meiosis [82]. Similarly, transcriptional activation of the majority of meiotic regulators also requires additional cues such as RA signalling. Aforementioned signalling networks highlight the need for nutrient sensing mechanism conservation in meiosis initiation all the way from single cell organisms to mammals.

## Epigenetic regulation of meiotic gene activation in mouse PGCs

To prepare for meiotic entry and acquire meiotic and developmental competence, global transcriptional changes are regulated differentially between the sexes. Transcriptional changes executed through global epigenetic reprogramming are most abrupt during the transition from PGCs to primary oocytes/spermatocytes [89,90]. This is evidenced by the global chromatin reorganisation and demethylation at E13.5 in mice which lead to large scale meiotic and embryonic gene activation [49]. At the chromatin level, removal of the Polycomb Repressive Complex 1 (PRC1) plays a crucial role in timely meiosis initiation [91]. The Polycomb Groups (PcGs) are chromatin remodellers responsible for transcriptional repression. PcGs contain different catalytic subunits allowing them to deposit repressive histone marks. The PRC1 central subunit, Rnf2, is a ubiquitin ligase responsible for inducing H2A ubiquitination. Rnf2 deletion gives rise to global reduction in UbH2A and de-repression of a subset of meiotic prophase genes such as *Stra8*, *Sycp3*, *Rec8* and *Hormad2* [91]. Moreover, meiotic initiator STRA8 is also a direct target of Rnf2 while PRC1 plays a role in antagonising RA signalling and suppressing STRA8 transcriptional activities in PGCs. Rnf2 depletion sensitises PGCs to RA signalling, indicating that down-regulation of PRC1-mediated transcriptional repression is a prerequisite to meiotic entry [91]. In fact, the concurrence of DNA demethylation and PRC1 erasure may be prerequisite for potentiating germline gene expression. Hill et al. identified a subset of genes that are activated during PGC epigenetic reprogramming which are referred to as the Germline Reprogramming Responsive (GRR) genes. These GRR genes were activated after dual depletion of 5mC and PRC1 in both male and female PGCs at E13.5 [92].

It is important to note that histone replacement occurs prior to meiotic entry during PGC development [93]. To establish specific histone modifications for meiotic gene activation, large scale chromatin remodelling via histone displacement takes place after global DNA methylation. Histone chaperones NAP1 disassemble and remove core linker histone H1, followed by extraction of H2AZ [94]. Consequently, core repressive marks such as H2A/H4R3me2, H3K9me3, H3K27me3 and H3K9ac are diminished at E11.5, resulting in a large proportion of open chromatin [93]. Notably, this mechanism is likely to be sex-specific. Ueda et al. [95] demonstrated that male-specific H3 variant H3t replaces H3.1 before entry into spermatogenesis and is more prone to inducing an open chromatin structure *in vitro*. These epigenetic mechanisms contribute to the priming of PGCs for meiotic gene activation.

## Future prospects in meiosis research

The challenge presented by the reductive and final nature of meiosis, and the impossibility of a true meiotic cell lines, has hampered our ability to easily manipulate individual components of the meiotic process. As a result, molecular mechanisms of meiosis have mostly been studied using singled cell organisms. However, although a number of pathways are conserved between the aforementioned model species, it is essential to understand specific mechanisms that are required for timely meiosis initiation among species, particularly in higher organisms. Therefore, mounting efforts have been invested into *in vitro* induction of meiotic entry utilising spermatogonial stem cells, induced pluripotent stem cells and embryonic stem cells, which can be differentiated into PGC-Like Cells (PGCLC) [96]. Hikabe et al. developed an *in vitro* culture system to induce meiotic entry and map the entire cycles of germ line maturation by co-culturing the murine PGCLCs with gonadal somatic cells. The resulting male and female mature gametes were fertile and gave rise to viable pups [97]. In addition, Hamazaki et al. [98] have successfully reconstituted the transcription networks required for oocyte maturation using mPGCLCs. In these PGCLCs, the essential transcription factors and epigenetic configurations required for meiotic initiation largely recapitulated those of mouse PGCs [99].

Although these models cannot fully replicate the *in vivo* conditions, they can be used to investigate environmental cues for meiotic entry. For example, Shimamoto et al. [100] reported that primary oocytes bypassed meiotic arrest at diplotene stage in prophase I without inducing hypoxia, indicating that hypoxia serves as one of the major factors maintaining oocyte dormancy which is mediated by nuclear localisation of FOXO3. Utilisation of these tools has led to recent appreciation of the evolutionarily conserved role of nutrient-deprivation in meiosis initiation which, combined with careful studies of meiosis-activating transcriptional networks, has led to landmark discoveries allowing experimental modelling of meiotic initiation in murine cells [78,87,97,98]. Now that meiosis initiation can be modelled and manipulated, the direct contribution of specific cellular process during meiosis initiation must be established.

## The role of nutrient sensing in meiotic initiation

In fission yeast, autophagy is likely to be an important player during meiotic initiation as it is known to be activated upon nutrient deprivation [101]. Autophagy is crucial for reserving energy in response to cellular stress conditions such as nutrient and oxygen starvation. In both fission and budding yeast meiotic entry fails if autophagy is deficient [102,103]. Autophagy acts to degrade major meiotic entry inhibitors allowing the cell to enter meiosis [104]. Furthermore, in *Drosophila,* even though nutrient starvation leads to a reduction in oocyte production, autophagy activation in follicle cells is essential for oogenesis due to its role in supporting signalling between nurse cells and oocytes [105]. Moreover, mouse models lacking an essential autophagy gene, AuTophaGy related 7 (Atg7), are infertile even though specific mechanism remains unclear [106,107]. However, in mammals STRA8 represses autophagy by binding the promoter of Nr1d1, which in turn leads to repressing its downstream target ULK1, an autophagy initiator, highlighting the requirement for the suppression of autophagy during meiosis initiation [108]. It is likely that metabolic stress, in concert with RA signalling, act to activate meiosis in mammals [108]. As mentioned in previous sections, Wang et al. [87] reported based on the scRNA-seq data that nutrient starvation is likely to be the metabolic stress inducing switching from glycolysis to mitochondrial oxidative phosphorylation. *In vivo*, this metabolic stress, which in the male is further contributed to by the blood-testis barrier (BTB), is required for SSC differentiation [109,110]. In female ovary, this stress could be created by germ cell cysts restricting nutrient access. Although the role for nutrient deprivation in meiosis is potentially conserved it is yet to be fully understood in mammals. It can be hypothesised that the suppression of autophagy might be important in maintaining meiotic DSBs during prophase I as autophagy plays a role in DNA damage repair [111]. The master regulator of autophagy, mTORC1, is found to be crucial during meiotic onset. The mTOR pathway is involved in many cellular processes such as cell growth, proliferation, protein synthesis, nutrient sensing and autophagy [112] with its role in meiotic entry largely conserved from yeast to mammals [113]. In both yeast and female drosophila, reduction in TORC1 expression in response to nutrient starvation is needed for mitotic-meiotic switch [114,115]. In *C. elegans*, stem cells proliferation and maintenance if the stem cell pool depend on autophagy via nutrient sensing pathways including TGFβ/DAF-7 signalling, ribosomal protein S6 kinase (S6K) and insulin IGF-1-like signalling (IIS) that are involved in TOR signalling [116–118]. However, the role of autophagy in meiotic entry in *C. elegans* is not well-explored. In mammals, however, TORC1 signalling is crucial for SSCs differentiation [119]. Suppression of mTORC1 activators is required for male mitotic arrest in PGCs [83], which could prime male germ cells for meiotic entry. Sahin et al. [120] demonstrated that mTOR is a target of RA signalling required for STRA8 expression during male meiotic onset. These observations suggest that in mammals, meiotic initiation requires mTOR1 expression and hence suppression of autophagy as these two factors are mutually exclusive. Consequently, metabolic stresses that acts on non-autophagic pre-meiotic cells, in parallel with RA stimulation, may be prerequisite for meiotic entry.

## Emerging links between meiotic initiation and oncogenesis

Recently uncovered links between nutrient deprivation and mammalian meiosis [87] raise a series of important questions. For many years, dogma stated that the Weismann barrier (a distinction between **‘**immortal' germ cell lineages that generate gametes and ‘disposable' somatic cells) explained the very tight control of meiotic gene expression, which was thought to limit transcription exclusively to germ cells. However, following technical advances in transcriptomic and proteomic analysis it is becoming increasingly clear that meiotic gene expression does occur in the soma, and in cancer cells. The first meiotic genes reported expressed in cancers were found to be immunogenic and were consequently classified as Cancer Testis Antigens (CTA), however with increasing number of CTAs identified it has become clear that these are not limited to testis specific proteins, and that not all of them are immunogenic. To address this issue, the term Germ Cell Cancer Genes (GCCG) has been proposed to describe the variety of meiotic genes re-expressed in cancer cells [121]. Currently over a thousand GCCGs have been reported widely re-expressed across cancer types, highlighting the importance of this process (see below) [105,121–124].

**Figure 2.**
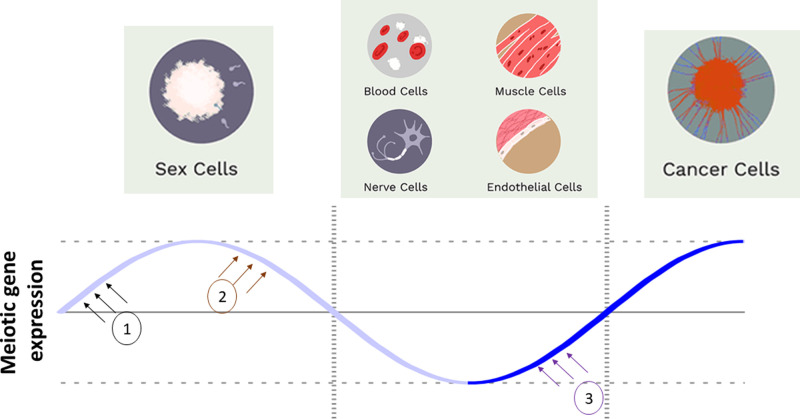
Expression of genes understood to be exclusively meiotic is assumed tightly regulated and silenced in healthy somatic cells. However, we still lack detailed mechanistic understanding of the machinery that turns on meiotic gene expression during meiosis initiation (**1**), turns expression off during the meiotic-to-mitotic transition (**2**), and (**3**) the factors responsible for aberrant reactivation of meiotic gene expression in, for example, cancer cells.

The presence of meiotic proteins in cells other than germ also brings into to question the validity of the Weismann barrier hypothesis [121,122,125], which now needs to be evaluated experimentally through a combination of cell signalling, epigenetic and transcriptional analyses. Indeed, it is now timely to consider whether genes required for meiosis specific processes might also possess non-meiotic functions; such ‘moonlighting’ functions have been characterised for a number of human mitotic proteins including the HSP family members, clathrin and dynein [126]. Recently, we identified a novel, ‘moonlighting’ function for the meiotic protein TEX12 (Testis Expressed 12) within microtubule organising centres, organelles that are fundamental for meiosis and mitosis [122]. TEX12 gene transcription and protein expression were originally believed to be exclusively meiotic with expressed protein restricted to the meiosis-specific synaptonemal complex, which assembles between aligned homologous chromosomes during prophase to facilitate DNA recombination. Similar moonlighting functions have been previously reported for other meiotic proteins such as Sme4 and Hei10 [127,128]. Furthermore, we found TEX12 to be a GCCG that is widely expressed in ∼15% of cancer patients [122]. Frequent expression of TEX12, and other meiotic genes in human cancers highlights that the process of oncogenesis might recreate, or be triggered by, some of the conditions characteristic of meiotic initiation resulting in large scale activation of the meiotic transcriptome. It appears possible, for example, that this might be linked to nutrient deprivation within solid tumours and associated changes in autophagy signalling It is also plausible that some meiotic genes are endogenously expressed in distinct cell and tissue types, for example in the brain, where the blood-brain barrier might recapitulate physiological processes that are known to activate meiosis in the testis, such as nutrient starvation experienced as a consequence of the blood-testis barrier (Figure 2). This is reminiscent of angiogenesis and shaping of the tumour microenvironment where nutrients are generally deprived [129]. In the tumour microenvironment, cancer cells require high nutrient uptake to maintain survival and proliferation during tumour initiation. mTOR complexes are known to play a role as amino acid sensors, allowing cancer cells to adapt to the harsh environment by epigenetic shift and metabolic reprogramming [130,131]. This recapitulates the amino acid restriction within the gonad that surrounds germ cells which could explain thereactivation of meiotic genes [87,132].

Notably, chromatin reorganisation can be affected by nutrient availability. Kirmes et al. [133] demonstrated that chromatin compaction and alteration of nuclear architecture can be induced by nutrient depletion. These changes could lead to down-regulation of meiotic gene suppressors during oncogenesis similarly to what happens at meiotic entry. In fission yeast, TORC1 signalling induces a facultative heterochromatin state at meiotic genes in a nutrient sensitive manner. Inhibition of TOR leads to disassembly of a heterochromatin islands forming complex [134]. Similar mechanisms may be exploited during meiotic gene re-activation in cancer, suggesting that global epigenetic changes play a role in meiotic gene activation during oncogenesis.

Collectively, metabolic stresses and mTOR signalling appear to be crucial for both meiotic gene expression and oncogenesis. Further reviews exploring the role of TOR pathways across different species and in cancer can be found here [113,130]. Emerging links between meiotic initiation and oncogenesis require further investigation and conserved meiotic entry pathways discussed in this review might provide novel insights to cancer initiation.

## Conclusion

**Figure 3.**
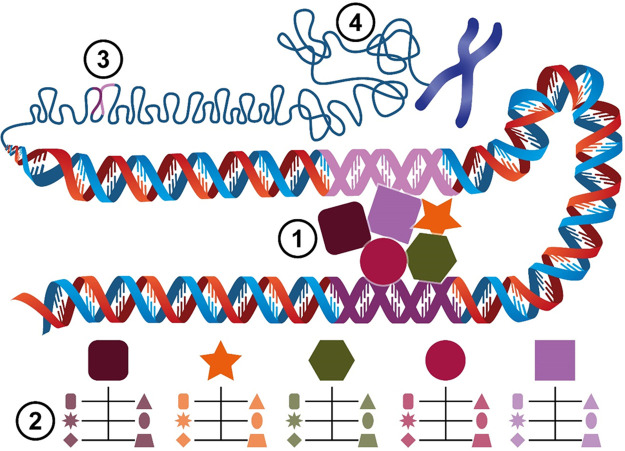
Unanswered questions about meiotic gene activation. (**1**) At the DNA level, interactions between the gene promoter (pink) and enhancer (purple) regions controls recruitment of transcription regulating machinery including transcription factors/repressors and their cofactors. What are the cell type specific meiotic promoter binding proteins and how do they regulate exclusively meiotic expression? (**2**) The availability of each meiotic promoter binding protein (shown as (**1**)) is affected by a network of specific cofactors (rectangle), kinases (star), phosphatases (diamond), E3s (triangle), deubiquitinating enzymes (oval) and others (trapezoid). What is the complete network of these meiotic expression master regulators? (**3**) Gene expression is affected by distal enhancers (purple) and the 3D genome architecture which controls their ability to interact with gene promoters (pink) to facilitate the recruitment of transcriptional machinery. What are the cell type specific distal enhancers of meiotic genes? (**4**) At a higher level of genome compaction large topologically associated domains (TADs) become the units of chromatin and a position of the gene within the TADs can affect its expression. What are the cell type specific TAD domain positions of meiotic genes and what role does the TAD positioning play in regulating the expression of meiotic genes?

Now that experimental models for murine meiotic initiation have been established, it is timely to revisit the Weismann barrier hypothesis in order to understand how concerted silencing of meiotic gene transcription in the autosomes is regulated and synchronised and also to define the contribution of germ transcriptome to the soma (Figure 3) under both normal and pathological conditions. This leads us to pose several key questions pertaining to DNA superstructure and the specific machinery used to ensure meiosis-specific transcription in response to a variety of cell signalling mechanisms (Figure 3). Understanding these processes will help drive a more complete understanding of the links between meiosis initiation, transcription and cancer.

## Open Access

Open access for this article was enabled by the participation of University of Liverpool in an all-inclusive *Read & Publish* pilot with Portland Press and the Biochemical Society under a transformative agreement with JISC.

## Abbreviations

BMP: bone morphogenic protein
CB: cytoblast
DTC: distal tip cell
FSH: follicle stimulating hormone
GB: gonialblast cell
GRR: germline reprogramming responsive
GSC: germline stem cells
IME1: Inducer of MEiosis 1
LH: luteinizing hormone
MPF: maturation promoting factor
PGCs: primordial germ cells
PKA: Protein Kinase A
PRC1: Polycomb Repressive Complex 1
RA: retinoic acid
RXR: retinoid X receptors
SSCs: spermatogonial stem cells
TORC1: Target Of Rapamycin Complex 1
